# Survival status and predictors of mortality among patients admitted to surgical intensive care units of Addis Ababa governmental hospitals, Ethiopia: A multicenter retrospective cohort study

**DOI:** 10.3389/fmed.2022.1085932

**Published:** 2023-02-02

**Authors:** Amanuel Sisay Endeshaw, Mulualem Sitot Fekede, Ashenafi Seifu Gesso, Esubalew Muluneh Aligaz, Senait Aweke

**Affiliations:** ^1^Department of Anesthesia, School of Medicine, College of Medicine and Health Sciences, Bahir Dar University, Bahir Dar, Ethiopia; ^2^Department of Anesthesia, School of Medicine, College of Health Sciences, Addis Ababa University, Addis Ababa, Ethiopia

**Keywords:** survival, predictors, mortality, intensive care unit, surgical patient, Addis Ababa

## Abstract

**Introduction:**

Critical care is a serious global healthcare burden. Although a high number of surgical patients are being admitted to the surgical intensive care unit (SICU), the mortality remained high, particularly in low and middle-income countries. However, there is limited data in Ethiopia. Therefore, this study aimed to investigate the survival status and predictors of mortality in surgical patients admitted to the SICUs of Addis Ababa governmental hospitals, Ethiopia.

**Methods:**

A multicenter retrospective cohort study was conducted on 410 surgical patients admitted to the SICUs of three government hospitals in Addis Ababa selected using a simple random sampling from February 2017 to February 2020. The data were entered into Epidata version 4.6 and imported to STATA/MP version 16 for further analysis. Bi-variable and multivariable Cox regression models were fitted in the analysis to determine the predictor variables. A hazard ratio (HR) with a 95% confidence interval (CI) was computed, and variables with a *p-*value <0.05 were considered statistically significant.

**Results:**

From a sample of 410 patients, 378 were included for final analysis and followed for a median follow-up of 5 days. The overall mortality among surgical patients in the SICU was 44.97% with an incidence rate of 5.9 cases per 100 person-day observation. Trauma (AHR = 1.83, 95% CI: 1.19–2.08), Glasgow coma score (GCS) <9 (AHR = 2.06, 95% CI: 1.28–3.31), readmission to the SICU (AHR = 3.52, 95% CI: 2.18–5.68), mechanical ventilation (AHR = 2.52, 95% CI: 1.23–5.15), and creatinine level (AHR = 1.09, 95% CI: 1.01–1.18) were found to be significantly associated with mortality in the SICU.

**Conclusion:**

The mortality of surgical patients in the SICU was high. Trauma, GCS <9 upon admission, readmission to the SICU, mechanical ventilation, and increased in the creatinine level on admission to the SICU were the identified predictors of mortality in the SICU.

## 1. Introduction

The surgical intensive care unit (SICU) is a surgical-specific unit that offers critical care to unstable patients with a variable disease process and a wide range of illness severity who require continuous monitoring and emergency intervention (1). Mortality in the intensive care unit (ICU) is a global public health concern, causing productivity and financial loss of approximately 1% of a country’s growth domestic product going toward financing for critical care (1).

Over the past few years, the number of critically ill surgical patients admitted to the ICU has increased, accounting for nearly one-third of all ICU admissions (2). Patients who had had general surgery, neurosurgery, orthopedic, and traumatology operations were the most common types of surgical patients admitted to the intensive care unit (3). Although a colossal groups of surgical patients are being admitted to the ICU in high-income nations, the mortality rate is meager, which has remained consistent and lower than 25% (2, 4–6). In contrast, up to 50% of surgical patients die in the ICUs in low-income countries (7, 8).

Age, length of stay, comorbidities, type of surgery, and condition on admission are the potential factors affecting the survival of surgical patients in the ICU (9, 10). Apart from clinical factors, the unavailability of most essential and specialized components of ICU plays the lion’s share of role in the poor outcome in low-resource settings (11). Even though surgical patients admitted to the ICU have a variable disease process and an extensive range of severity of illness contributes to poor outcomes (9, 10), studies regarding risk factors for mortality in the SICU are sparse in low-resource settings, including Ethiopia, which hinders practical adjustment to improve outcome. Studies conducted to determine predictors of mortality among surgical patients admitted to the SICU are limited to high-income countries and depend on staff availability, clinical and laboratory indices which are not readily available in low-resource settings where poor outcomes are reported despite the greater burden of critical diseases (11).

There is a dearth of research conducted on surgical patients in the critical care context in Ethiopia and mortality predictors of surgical patients admitted to the ICU have not been determined. In addition, published data in low-resource settings did not thoroughly report the predictors of mortality among surgical patients admitted to the ICU.

For healthcare professionals, the early identification of patients who are more likely to survive and benefit may help to make informed decisions regarding patient care (12). Determining predictors of mortality among surgical patients admitted to the ICU will be vital to forward resources to the best practice based on the evidence-based prediction of patient outcomes. Therefore, this study aimed to estimate the incidence of mortality and identify predictors among surgical patients admitted to the ICUs of governmental hospitals in Addis Ababa, Ethiopia. This study will help health professionals to providing information about factors associated with mortality in the SICU and maximize their effort in preventing the problem. This study will also help to assess the quality of critical care delivery at the study hospitals in Addis Ababa, Ethiopia.

## 2. Methods and materials

### 2.1. Study design, period and setting

This multicenter retrospective cohort study was conducted from February 1, 2017, to February 29, 2020 in three governmental hospitals in Addis Ababa: Tikur Anbessa Specialized Hospital (TASH), Saint Paul’s Hospital Millennium Medical College (SPHMMC), and Saint Peter Specialized Hospital (SPSH). These hospitals were chosen purposely because they are the largest and teritiary specialized hospitals with relatively higher number of ICU beds, providing the majority of surgical patient care and admissions to the SICU across the country.

This study is reported according to the STROBE guideline for cohort study (13).

### 2.2. Population and eligibility criteria

The source population was all surgical patients admitted to the ICUs of selected governmental hospitals from February 2017 to February 2020. The study population was surgical patients admitted to SICUs of selected hospitals who met the inclusion criteria and selected for the sample during the study period.

All adult surgical patients above 18 years old admitted to the ICU of the selected hospitals between February 1, 2017 to February 29, 2020, were included. Pediatrics patients and adult patients with incomplete records, such as survival status information (death or censored), medical records without the admission and discharge date to and from the SICU were excluded.

### 2.3. Study variables

The primary outcome variable was time to death in the SICU (event), and the explanatory variables were sociodemographic factors, clinical and physiologic factors: surgical category, readmission, coexisting illness, trauma, cancer, laboratory results at admission, vital signs at admission, interventions did and complicatons at the SICU.

### 2.4. Sample size and sampling techniques

The sample size was determined based on the sample size calculation formula for survival analysis, taking *α* = 0.05, *β* = 0.2, SD = 0.5, HR = 1.92 from a Yemen study (14). Assuming the probability of an event = 0.2 and withdrawal = 0.1 after adjusting for censoring and withdrawal, the event was calculated to be 74; then the final sample size was 410.

**Figure 1:**
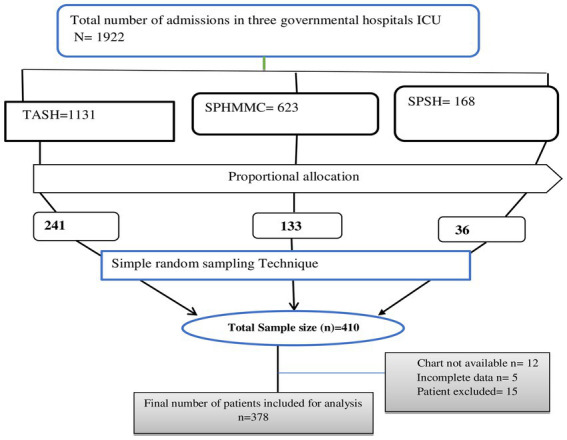
Flow diagram of proportional allocation and sampling of study participants.

Study subjects for each hospital were selected using a proportion allocation algorithm by dividing the number of admissions in each hospital SICU multiplied by the sample size (*n* = 410) by the total number of admissions in the three SICU hospitals (*N* = 1922). Study participants from each ICU were selected using a simple random sampling technique using the card number of patients from the logbook registry as a sampling frame who were admitted during the study period (Figure 1).

### 2.5. Data collection and quality control

After a systematic review of literatures, a data extraction checklist was prepared in English. Two BSc anesthetists collect the data under the supervision of one MSc anesthetist at the selected hospitals SICU. The data clerks also support them by identifying the cards of patients. Ethical clearance was obtained from the Institutional Review Board (IRB) of the College of health sciences Addis Ababa University (reference number: Anes/3 /2021/2022). Since this is a secondary data, informed permission was not required from each client; instead, it was sent to the head of the respective hospital’s management. Then, data collection begun after ethical clearance was obtained from the study hospitals.

To ensure data quality, a pre-test was conducted on 5% of the calculated sample size at Menelik II Comprehensive Specialized Hospital, and corrections were made accordingly. Training on the study objective and how to review the documents per the data extraction format was given to data collectors and supervisor for 1 day before data collection.

### 2.6. Data processing and analysis

Data were checked for completeness, coded, entered and cleaned using EpiData version 4.6. Then, data were exported to STATA/MP Version 16 for analysis. Missing values and outliers were checked by using explorative data analysis. Multicollinearity was checked using variance inflation factor (VIF) with a tolerance of 10, and the mean VIF was 1.78. Normal data distribution was checked by the Q-Q plot. Mean and standard deviation for normally distributed data and median for non-normally distributed data were used for continuous variables. Frequency and percentages were used to describe categorical variables.

The Kaplan–Meier failure curve and the log-rank test were fitted to test for the presence of a difference in death among the categorical variables. Categorical variables with *p-*value <0.05 on the log-rank test were considered to be fitted in the Cox regression model based on the assumption of the Cox regression. Before fitting the Cox regression model, data fitness and proportional hazard assumptions were evaluated using the log–log plot [log (−log (survival probability) *Vs.* log (survival time) and Schoenfeld residuals test)]. A bivariate Cox regression analysis was performed to measure the effects of each independent variable on the dependent variable. Variables with a *p-*value of <0.2 in the bivariable Cox regression analysis were entered for multivariable Cox regression analysis to identify the independent predictors of mortality. At a *p*-value of <0.05, all statistical tests were considered significant.

### 2.7. Operational definitions

*Survival status*: The outcome of a surgical patient admitted to ICU either event or censored.

- *Event*: Death in the SICU.
- *Censored*: Surgical patient admitted to the SICU but recovered and discharged to wards, and lost to follow-up.

*Loss to follow up*: Patients who were referred to other hospital or discharged against medical advice without knowing their outcome.

*Incomplete records*: Patient’s medical records without the admission date to the SICU, the discharge date from the SICU and survival status information.

*Readmission*: Patients who were readmitted to the SICU in the same hospital stay.

*Anemia*: Hemoglobin levels <12 g/dl in women and < 13 g/dl in men (15).

*Complications at ICU*: Diseases developed during stay in the SICU which is not reported upon admission such as cardiac arrest, anemia, arrhythmia, infection, hypotension, hypertension, aspiration etc.

## 3. Results

### 3.1. Socio-demographic and clinical characteristics of participants

From a sample of 410 patients, 378 were included in this study for final analysis, for a response rate of 92.2%. Thirty two study subjects were excluded from the study: five had incomplete data, 12 had lost charts, and 15 were pediatrics. Of the total sample, about half (50%) of patients admitted to the SICU were older than 33 years (Table 1).

**Table 1:** Socio-demographic and clinical characteristics of surgical patients admitted to ICU of hospitals in Addis Ababa, February 2017 – February 2020.

Variable	Category	Event (*n* = 170)	Censored (*n* = 208)	Total (*n* = 378)
		*n* (%)	*n* (%)	*n* (%)
Age in years (14)	≤33	80 (47)	109 (52.4)	189 (50)
	>33	90 (53)	99 (47.6)	189 (50)
Sex	Female	71 (41.77)	83(40)	154 (40.7)
	Male	99 (58.23)	125(60)	224 (59.3)
Residence	Rural	71 (41.77)	97 (46.64)	168 (44.44)
	Urban	99 (58.23)	111 (53.36)	210 (55.56)
Systolic blood pressure (mmHg)	<90	84 (49.41)	82 (39.42)	166 (43.92)
	90–140	70 (41.18)	112 (53.85)	182 (48.15)
	>140	16 (9.41)	14 (6.73)	30 (7.94)
Pulse rate (beats per minute)	<60	26 (15.29)	12 (5.77)	39 (10.06)
	60–100	81 (47.65)	142 (68.27)	223 (58.99)
	>100	63 (37.06)	54 (25.96)	117 (30.95)
Respiratory rate (breaths/min)	Normal	98 (57.65)	164 (78.85)	262 (69.31)
	Tachypnea	72 (42.35)	44 (21.15)	116 (30.69)
SpO2 (%)	<90	58 (34.12)	19 (9.13)	77 (20.37)
	≥90	112 (65.88)	189 (90.87)	301 (79.63)
Temperature (degree celsius)	<36.5	76 (44.71)	61 (29.33)	137 (36.24)
	36.5–37.5	68 (40.00)	137 (65.87)	205 (54.23)
	>37.5	26 (15.29)	10 (4.81)	36 (9.52)
GCS	<9	103 (60.59)	8 (3.85)	111 (29.37)
	9–12	28 (16.47)	12 (5.77)	40 (10.58)
	13–15	39 (22.94)	168 (80.77)	207 (54.76)
WBC (1,000 cell/mm^3^)	<4.5	8 (4.71)	7 (3.37)	15 (3.97)
	4.5–11	97 (57.06)	142 (68.27)	239 (63.23)
	>11	65 (38.24)	59 (28.37)	124 (32.80)
Anemia	Yes	100 (58.82)	87 (41.83)	187 (49.47)
	No	70 (41.18)	121 (58.17)	191 (50.53)
Platelet (1,000 cell/mm^3^)	<100	13 (7.65)	14 (6.73)	27 (7.14)
	≥100	157 (92.35)	194 (93.27)	351 (92.86)
SGOT (U/L)	≤32	114 (67.06)	148 (71.15)	262 (69.31)
	>32	56 (32.94)	60 (28.85)	116 (30.69)
SGPT (U/L)	≤32	120 (70.59)	154 (74.04)	274 (72.49)
	>32	50 (29.41)	54 (25.96)	104 (27.51)
Sodium (mmol/l)	<135	17 (10.00)	10 (4.81)	27 (7.14)
	135–145	144 (84.71)	193 (92.79)	337 (89.15)
	>145	9 (5.29)	5 (2.40)	14 (3.70)
Potassium (mmol/l)	<3.5	25 (14.71)	13 (5.77)	37 (9.79)
	3.5–5	135 (79.41)	192 (92.31)	327 (86.51)
	>5	10 (5.88)	3 (1.44)	13 (3.44)
Admission frequency	First time	140 (82.35)	201 (96.63)	341 (90.21)
	Readmitted	30 (17.65)	7 (3.37)	37 (9.79)
Mechanical ventilation	Yes	158 (92.94)	46 (22.12)	204 (53.97)
	No	12 (7.06)	162 (77.88)	174 (46.03)
Vassopressor use	Yes	115 (67.65)	45 (21.63)	160 (42.33)
	No	55 (32.35)	163 (78.37)	218 (57.67)
Complication	Yes	137 (80.59)	23 (11.06)	160 (42.33)
	No	33 (19.41)	185 (88.94)	218 (57.67)
Creatinine (mg/dl), Median (IQR)		1.5 (1.0, 1.9)	0.6 (0.5, 0.9)	0.8 (0.6, 1.5)

*p*, frequency; %, percentage; SpO2, peripheral oxygen saturation; mmHg, millimetre of mercury; WBC, white blood cell; SGOT, serum glutamic-oxaloacetic transaminase; SGT, serum glutamic pyruvic transaminase.

In this study, the most common reason for admission to the SICU was after surgery for postoperative follow-up (49.5%) followed by traumatic brain jury (15%), sepsis (12.6%), hemorragic shock (8.8%), acute respiratory failure (8.5%), and burn (5.6%). After admission to the SICU, more than half (53.97%) patients were on mechanical ventilator, 42.33% were on vasopressor support, 14.55% were on nasogastric tube feeding, 25% received blood components, 2.65% underwent dialysis, and tracheostomy was done in 5.29% of patients.

Nearly 50% of patients (49.5%) were anemic, 17.7% were hypoxic, with SpO_2_ below 90, and 43.9%, 30.95 and 29.4% patients had Systolic blood pressure < 90 mmHg, pulse rate > 100 beats/min, and GCS score < 9, respectively. The median with interquartile range (IQR) for creatinine level upon admission to the SICU was 0.8 (0.6–1.5) mg/dl (Table 1).

Furthermore, after admission to the SICU, 160 (42.33%) patients developed some form of complication in the SICU. Cardiac arrest, infection, and arrhythmia were the most common complications.

### 3.2. Survival status of patients admitted to the SICU

In this study, patients were followed for a minimum of 4 h to a maximum of 41 days, with a median follow-up of 5 days (IQR = 2–8). Based on this, the total person-time observation was 2636.7 person-days. In this study, 170 (44.97%) surgical patients died in the ICU. About 208 (55.03%) surgical patients admitted to the ICU were censored: 196 (51.85%) discharged alive, 9 (2.38%) left against medical advice, and 3 (0.79%) referred to other hospitals.

**Figure 2:**
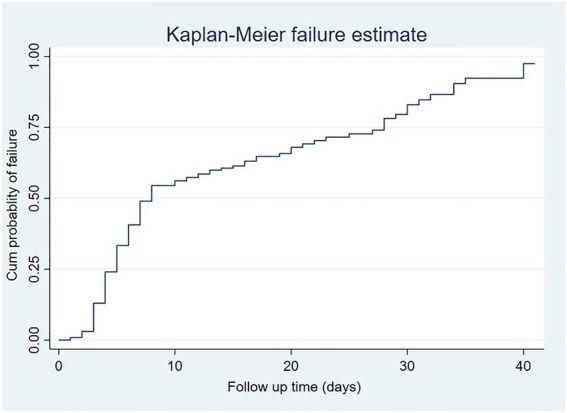
A Kaplan–Meir failure estimate of surgical patients admitted to ICU from February 2017 to February 2020.

The incidence of death among surgical patients in the ICU was found to be 6.3 cases (95% CI, 5.4**–**7.3) per 100 person-day observation. The cumulative probability of failure on the first day of admission to ICU was 0.81%, at the third to fifth days was 32%, and the sixth to tenth and the end of the follow-up was 95.4%. The median survival time was found to be 8 days (Figure 2).

### 3.3. Predictors of mortality among surgical patients admitted to ICU

Based on the log-rank test, there was a statistically significant difference in survival among categories of age, Glasgow coma scale (GCS), trauma, coexisting illness, SpO_2_, mechanical ventilation, frequency of admission, vasopressor use, and complication in the SICU (*p* < 0.05). However, there was no significant difference among categories of most physiologic factors, such as heart rate.

After all covariates and the whole model satisfied the proportional-hazards assumption based on the global proportional-hazards test using the Schoenfeld residuals [*X*^2^ (10) = 13.37, *p* = 0.2036], data were fited into the Cox regression model. Linearity assumption was also fulfilled for the creatinine level, included in the multivariable Cox model.

All the variables, which were entered into bivariable Cox regression analysis, had *p*-values of <0.2 and they were fitted into multivariable Cox regression analysis. Then, on a multivariable Cox regression analysis, it was found that only trauma, frequency of admission, GCS score, mechanical ventilation, and creatinine level at admission to the SICU were identified as significant predictors for time to death among surgical patients admitted to the ICU (*p* < 0.05) (Table 2).

**Table 2:** Multivariable Cox regression analysis for mortality predictors among surgical patients admitted to the SICU, Addis Ababa, Ethiopia, February 2017 – February 2020.

Variable	Category	Survival status		Crude HR	Adjusted HR
		Event	Censored	(95% CI)	(95% CI)
Age in years	≤33	80	109	1	1
	>33	90	99	1.24 (0.91, 1.67)	1.15 (0.83, 1.59)
Trauma	Yes	125	18	4.41 (3.14, 6.25)	1.83 (1.19, 2.80)**
	No	45	190	1	1
Coexisting illness	Yes	69	3	2.53 (1.86, 3.44)	1.04 (0.73, 1.48)
	No	101	205	1	1
Admission frequency	First time	140	201	1	1
	Readmitted	30	7	4.91 (3.18, 7.58)	3.52 (2.18, 5.68)***
Glasgow Coma Scale	<9	103	8	5.11 (3.52, 7.42)	2.06 (1.28, 3.31)**
	9–12	28	32	2.75 (1.69, 4.49)	1.70 (1.01, 2.85)*
	13–15	39	168	1	1
SPO_2_ (%)	<90	48	19	1.58 (1.11, 2.18)	1.09 (0.77, 1.54)
	≥90	122	189	1	1
Mechanical ventilation	Yes	158	46	6.64 (3.68, 11.96)	2.52 (1.23, 5.15)*
	No	12	162	1	1
Vasopressor administered	Yes	115	45	1.82 (1.31, 2.54)	0.80 (0.55, 1.16)
	No	55	163	1	1
Creatinine (mg/dl)				1.14 (1.08, 1.21)	1.09 (1.01, 1.18)*
Complication	Yes	137	23	1.69 (2.51, 5.41)	1.33 (0.84, 2.10)
	No	33	185	1	1

1, reference group; HR, Hazard ratio; CI, confidence interval; ***p-value < 0.001; **p-value < 0.01; *p-value < 0.05.

Keeping all other variables constant, patients with traumatic injury had an 83% higher mortality hazard than those without traumatic injury (AHR = 1.83, 95% CI:1.19, 2.80). The hazard of mortality among surgical patients in the SICU with a severe brain injury (GCS <9) was more than two times than those patients with a mild brain injury (GCS 13–15) (AHR = 2.06, 95% CI: 1.28, 3.31). Similarly, patients with a moderate brain injury (GCS 9–12) had a 70% higher risk of death than those with a mild brain injury (AHR = 1.70, 95% CI: 1.01, 2.85).

The mortality hazard for readmitted patients to the SICU was 3.52 times higher than the hazard of patients admitted for the first time (AHR = 3.52, 95%CI: 2.18, 5.86). In addition, the hazard of death for patients on mechanical ventilator during their SICU stay was 2.52 times higher than their counterparts (AHR = 2.52, 95% CI:1.23, 5.15). Furthermore, upon admission to the SICU, a one-unit increase in the patients’ creatinine level increased the mortality hazard of patients by 9% (AHR = 1.09, 95% CI:1.01, 1.18) (Table 2).

## 4. Discussion

This study primarily investigated the incidence and predictors of mortality among surgical patients hospitalized in ICUs of three governmental hospitals in Addis Ababa, Ethiopia. Using a survival analysis model, our investigation assessed the patients’ sociodemographic, clinical, and physiologic variables from their medical record data. Most previous studies recorded the mortality among surgical patients admitted to ICU were cross-sectional in design and used logistic regression and descriptive approaches, limiting the identification of predictors. Consequently, characteristics such as trauma, being readmitted to the SICU, low GCS score, and admission creatinine level were identified to be significantly associated with mortality in the SICU in our study.

The overall incidence of mortality in the SICU of three hospitals in Addis Ababa was found to be 44.97%. This result is consistent with the findings of studies done in Pakistan, Rwanda, Nigeria, Saint Paul Hospital Millennium Medical College, and Jimma University Specialized Hospital (16–20). However, it was higher than the previous study done in Tikur Anbessa Specialized Hospital (21). This contradiction might be due to a difference in the study design and population.

Our result is also higher than studies conducted in most high and middle-income countries, including Ireland, China, Brazil, Thailand, and South Africa (4, 8, 9, 22, 23). This discrepancy might have resulted from the shortage of essential medications and equipment, which could significantly affect survival from the SICU (24, 25). Our result was also higher than those of low-income countries, such as Yemen and Kenya (14, 26). A difference in follow-up and sample size might explain this inconsistency.

In our study, patients with traumatic injuries were shown to have an elevated mortality risk in the SICU. This finding is similar to a study conducted in Brazil, which demonstrated that trauma increases the risk of patients’ mortality treated in the SICU (27). The possible reason could be that patients who sustained trauma are more likely to develop respiratory failure, hemorrhagic shock, and acute kidney injury than non-trauma patients (28).

In this study, a low GCS significantly contributed to the death of surgical patients admitted to ICU. This result is consistent with a study conducted by Pogorzelsk et al. (23). Low GCS score is linked with traumatic brain injury in which patients can not protect their airways, so the high risk of aspiration and aspiration pneumonia could negatively influence survival (29, 30). Another explanation could be that patients with a low GCS have a risk of hypoxia due to compromised airway patency, increasing mortality risk in the SICU. In addition, cerebral hypoxia from hypotension of a decompensated disease process is a sign of multiorgan failure where survival will be significantly affected (30).

Our study also revealed that patients who were readmitted to the SICU had a significantly higher mortality risk compared to patients admitted for the first time. This result is in line with studies done in America, Sweden, and Germany (31–34). The reason to explain this finding could be readmitted patients might have a higher severity of illness, which will reduce the physiologic reserve and increase the risk of organ failure, increasing the chance of mortality. In addition, reoperation is more prevalent among readmitted patients which could lead to a deterioration in the patient’s immunological function, increasing the risk of hospital-acquired infection and sepsis so that the patient’s chance of survival in the SICU will be reduced (32, 35). Furthermore, due to the lack of formal SICU discharge guidelines in the study areas, premature discharge of a patient might mask the untreated pathological organ damage, leading the patient to readmission and further increasing organ failure in the SICU.

Surgical patients who were on mechanical ventilation had a high risk of mortality than patients who were not on mechanical ventilation. This finding is in line with studies done Ethiopia, Kenya, and Brazil (16, 36, 37).This could be due to patients on mechanical ventilator will be immobile for an extended period, increasing the chance of venous thromboembolism, and they are susceptible in developing ventilator-associated pneumonia, which could decrease the chance of survival (38, 39).

Our study also showed that creatinine level increment would affect surgical patients’ survival in the ICU. A single unit increment of creatinine increasd the mortality risk by 9% despite sensitivity and specificity to predict mortality using creatinine. Our result is consistent with a study done by Samuels et al. (40). The reason could be an increased creatinine level indicates a kidney injury resulting in loss of function in which metabolism is greatly affected, which leads to electrolyte imbalance and metabolic acidosis that greatly impacts mortality (41). In addition, patients with a compromised kidney function noted by increased creatinine levels might have associated comorbid conditions and sepsis, which could reduce the likelihood of survival (42, 43). Another reason might be that patients with some form of kidney injury are at an increased risk of developing lung and hepatic injury, which results in respiratory and liver failure, reducing the survival (44).

### 4.1. Strength of the study

The study was conducted in a multicenter setting using random sampling and patients were followed for a long duration with a maximum of 41 days, increasing the study’s generalizability.

### 4.2. Limitations of the study

Because the study design was retrospective, some significant predictors, such as body mass index, were unavailable. Furthermore, physiologic scores such as acute physiological and chronic health evaluation (APACHE) and sequential organ failure assessment (SOFA) were not obtained, which could have influenced mortality.

## 5. Conclusion

In this retrospective cohort study, we found that the overall mortality among surgical patients admitted to the ICUs of three governmental hospitals in Addis Ababa was high. Trauma, low GCS score, readmission to SICU, mechanical ventilation, and creatinine level were the significant risk factors for surgical patients mortality in the ICU.

Therefore, protocols for discharge of patients from the ICU must be considered to minimize readmission, detection and proper management of patients with elevated creatinine levels upon admission to the ICU must be employed. We recommend large-scale studies with a prospective design including nutritional determinants and severity scores such as the APACHE and SOFA to better understand mortality and its predictors among surgical patients in the ICU.

## Data availability statement

The original contributions presented in the study are included in the article/Supplementary material, further inquiries can be directed to the corresponding author.

## Ethics statement

The studies involving human participants were reviewed and approved by the Institutional Review Board (IRB) of the College of Health Sciences Addis Ababa University (reference number: Anes/3 /2021/2022). Written informed consent from the participants was not required to participate in this study in accordance with the national legislation and the institutional requirements.

## Author contributions

MF and AE contributed to the study conception and design, performed the statistical analysis and interpretation of the results, and wrote and prepared the manuscript. AG, EA, and SA contributed to the study conception, design, data collection, writing up, statistical analysis and interpretation of the results. All the authors read the manuscript and approved the final submission.

## Funding

This work was funded by Addis Ababa University. The funder had no role in study design, data collection and analysis, decision to publish, or preparation of the manuscript.

## Conflict of interest

The authors declare that the research was conducted in the absence of any commercial or financial relationships that could be construed as a potential conflict of interest.

## Publisher’s note

All claims expressed in this article are solely those of the authors and do not necessarily represent those of their affiliated organizations, or those of the publisher, the editors and the reviewers. Any product that may be evaluated in this article, or claim that may be made by its manufacturer, is not guaranteed or endorsed by the publisher.

## Abbreviations

AHR: adjusted hazard ratio
APACHE: Acute Physiology and Chronic Health Evaluation
BSc: Bachelor of science
GCS: Glasgow Coma Scale
ICU: intensive care unit
MSc: Master of science
SICU: surgical intensive care unit
SOFA: sequential organ failure assessment
