# Supplementary material for: Survival status and predictors of mortality among patients admitted to surgical intensive care units of Addis Ababa governmental hospitals, Ethiopia: A multicenter retrospective cohort study
Source: Front Med (Lausanne). 2023 Feb 2;9:1085932. doi: 10.3389/fmed.2022.1085932 (PMC9932811; doi:10.3389/fmed.2022.1085932)
Supplement: Supplementary file 1 [file Data_Sheet_1.PDF]

Table 1. Multicollinearity test using VIF for independent variables

Variable	VIF	1/VIF
Age	1.07	0.935637
Trauma	1.89	0.528810
Creatinine	1.21	0.825398
SpO2	1.08	0.926505
Mechanical ventilation	3.05	0.327909
Vasopressor	1.51	0.660626
Frequency of admission	1.12	0.889499
Complication	2.47	0.404832
Coexisting illness	1.37	0.727504
GCS	2.18	0.459128
Mean VIF	1.78	

Table 2. Log rank test for all categorical variables to predict surgical ICU mortality

Variables	df	Chi-square	P-value
Gender	1	0.02	0.8839
Residence	1	0.39	0.5300
Age	1	4.60	0.0320
Surgical category	2	4.20	0.1225
Trauma	1	80.76	0.0000
Cancer	1	1.53	0.2168
Coexisting illness	1	36.27	0.0000
Frequency of admission	1	68.46	0.0000
Mechanical ventilation	1	36.27	0.0000
Vasopressor	1	12.28	0.0005
Heart rate	2	14.35	0.1245
SBP	2	4.85	0.0886
SpO2	1	6.29	0.0121
Respiratory rate	1	2.48	0.1151
Temperature	2	5.38	0.0678
GCS	2	85.39	0.0000
WBC	2	1.77	0.4130
Anemia	1	0.89	0.3468
PLT	1	0.17	0.6786
SGOT	1	1.00	0.3178
SGPT	1	0.65	0.4189
Sodium	2	8.34	0.2345
Potassium	2	3.07	0.2157
Complication	1	48.73	0.0000

Figure 1. log-log plot by trauma, vasopressor age, and mechanical ventilation

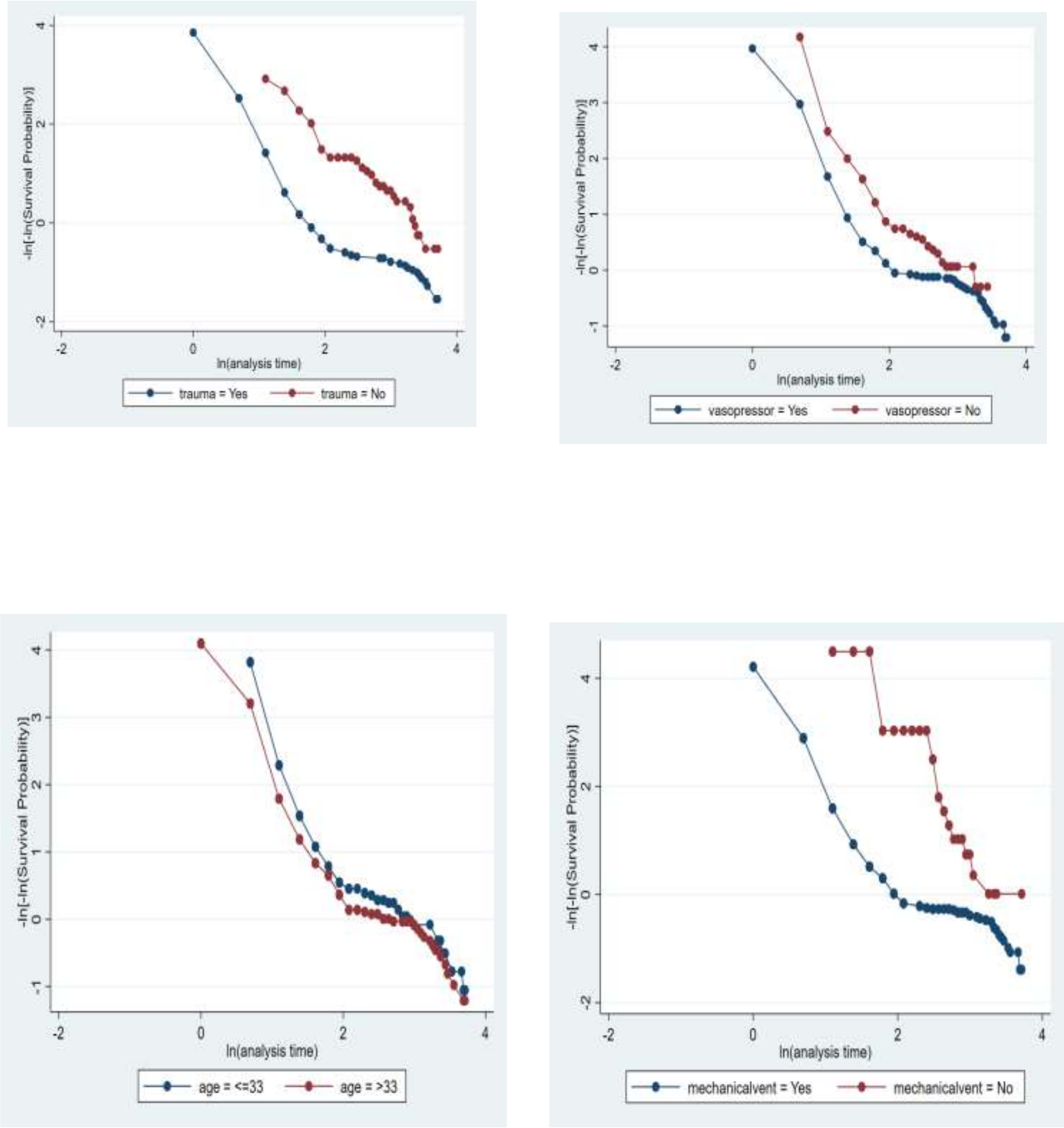

Figure 2. log-log plot by GCS, SpO2, and frequency of admission

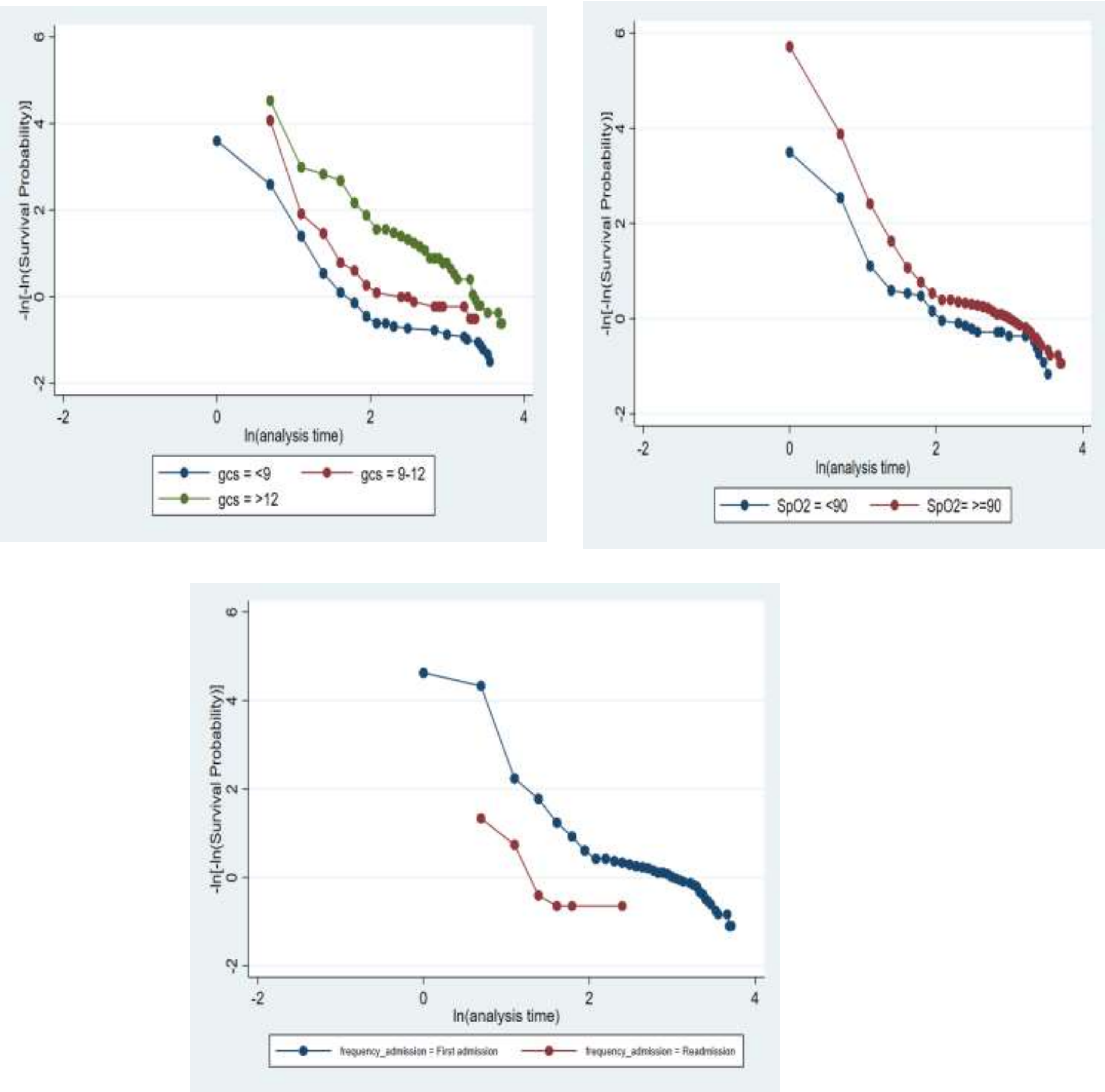

Figure 3. Kaplan Meir failure estimate by frequency of admission, GCS, mechanical ventilation and SpO2

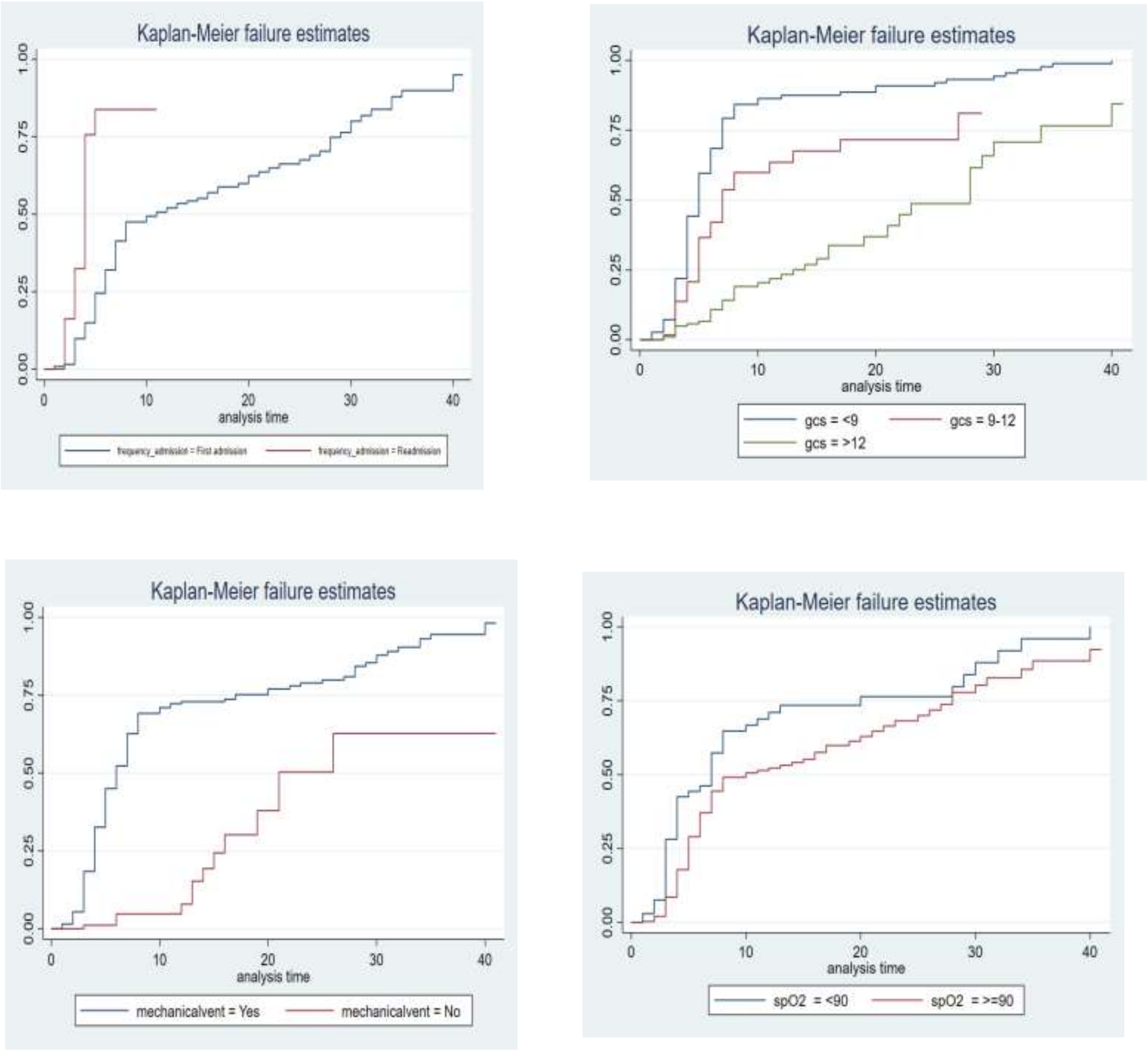

Figure 4. Kaplan Meir failure estimate by age, vasopressor, coexisting and complication

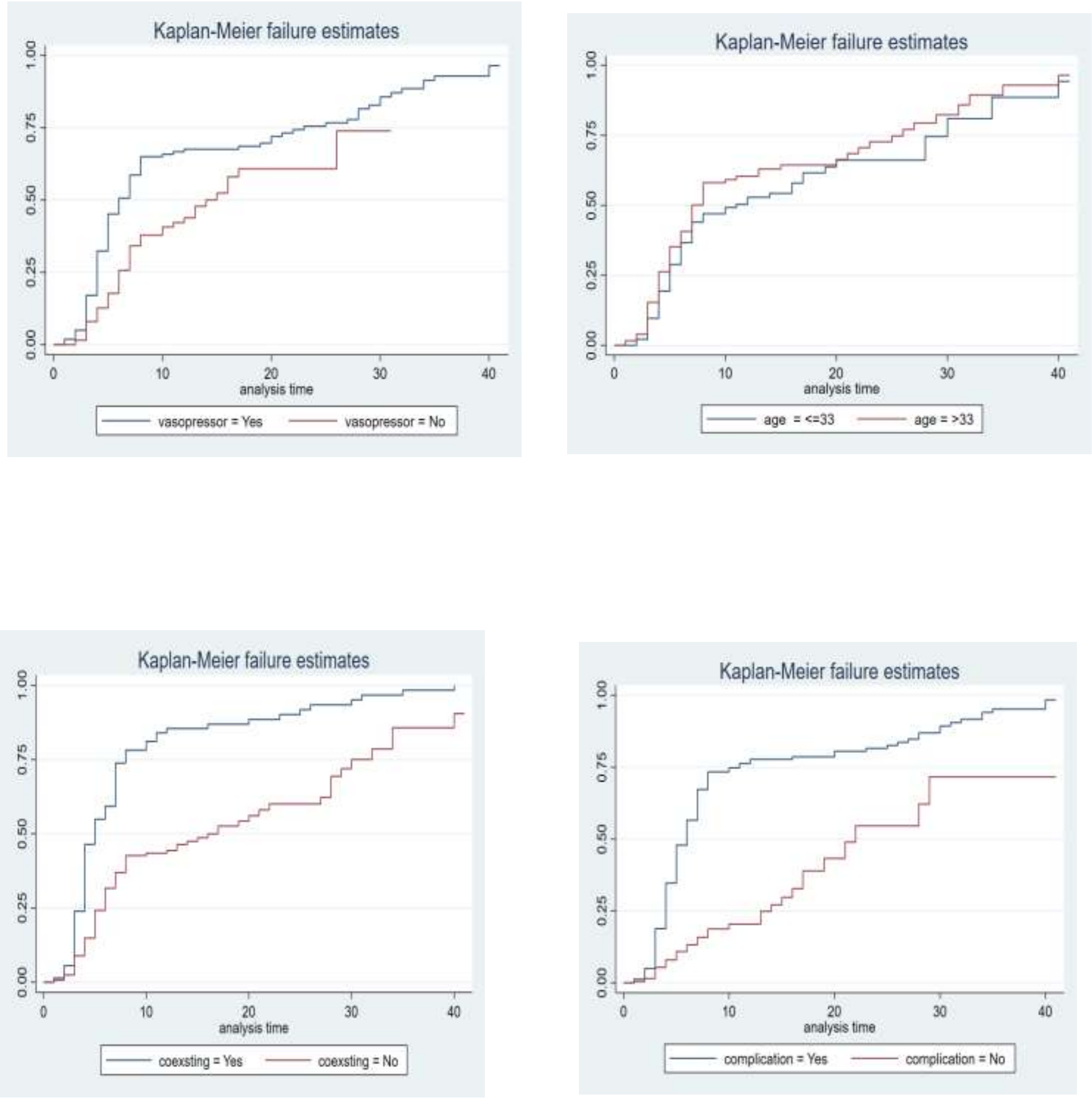

Table 3. Schoenfeld residual

Variable	rho	Chi^2	df	Prob>chi^2
Age	-0.05178	0.45	1	0.5010
Trauma	0.10710	2.53	1	0.1116
Creatinine	0.7454	0.63	1	0.4289
SpO2	-0.00405	0.00	1	0.9577
Mechanical ventilation	0.09673	1.61	1	0.7525
Vasopressor	0.01278	0.03	1	0.8555
Frequency of admission	0.02718	0.13	1	0.7164
Complication	0.03574	0.25	1	0.6180
Coexisting illness	0.02274	0.10	1	0.7525
GCS	0.01818	0.07	1	0.7951
Global test		13.37	10	0.2036
